# Dystonia: Still a Mysterious Syndrome

**DOI:** 10.3390/life12070989

**Published:** 2022-07-04

**Authors:** Ryoma Morigaki, Ryosuke Miyamoto

**Affiliations:** 1 Department of Advanced Brain Research, Institute of Biomedical Sciences, Graduate School of Medicine, Tokushima University, 3-18-15 Kuramoto-Cho, Tokushima 770-8503, Tokushima, Japan; 2 Department of Neurosurgery, Institute of Biomedical Sciences, Graduate School of Medicine, Tokushima University, 3-18-15 Kuramoto-Cho, Tokushima 770-8503, Tokushima, Japan; 3 Parkinson’s Disease and Dystonia Research Center, Tokushima University Hospital, 2-50-1 Kuramoto-Cho, Tokushima 770-8503, Tokushima, Japan; ryom@tokushima-u.ac.jp; 4 Department of Neurology, Institute of Biomedical Sciences, Graduate School of Medicine, Tokushima University, 3-18-15 Kuramoto-Cho, Tokushima 770-8503, Tokushima, Japan

The diagnosis of dystonia is sometimes complicated due to its many clinical manifestations, causes, and the lack of specific diagnostic examinations or simple algorithms [1,2,3,4]. In this Special Issue, Biase et al. updated a dystonia classification algorithm based on phenomenology, which could be useful to movement disorder specialists [5]. Although recent developments in AI-based deep learning technology offer highly accurate diagnostic tools in neuroimaging [6], clinical phenomenology, mainly motor manifestations, remains the gold standard for diagnosing dystonia.

The importance of clinical phenomenology in the diagnosis of dystonia is similar to that of psychiatric diseases. Interestingly, dystonic movements in Oppenheim’s dystonia were thought to be psychogenic before the discovery of the *DYT1 (Tor1A)* gene [7,8]. Currently, dystonia is recognized as an organic disease, mainly due to dysfunction of the brain motor circuit; however, psychiatric comorbidities are often discussed [9]. From a psychiatric point of view, functional neurological disorders, including functional dystonia, a psychiatric disease defined in the DSM-5, make it difficult to distinguish organic dystonia from psychiatric disorders [10]. For discrimination purposes, a highly accurate diagnostic algorithm based on the phenomenological features of functional dystonia was developed [11], and a neuroimage-based diagnostic tool was investigated [12,13,14]. These attempts have uncovered the pathogenesis of functional dystonia, which might also be a brain circuit disorder. However, similar to organic dystonia, phenomenological features are still the gold standard for diagnosing functional dystonia, and the personality profiles in patients with dystonia seem to be unique, novel, and important [15]. Regardless of its significance, the personality profile is largely unknown and might be important for clinicians considering treatment strategies.

In addition to medication therapy, pallidal deep brain stimulation (DBS) surgery is a promising treatment for medically intractable primary dystonia, which involves the cortico–basal ganglia–thalamocortical motor loop in its pathogenesis [16,17]. Jamora et al. showed the efficacy of lesioning the pallidothalamic tract using focused transcranial MR-guided ultrasound in dystonia patients with DYT3/XDP-TAF1, suggesting the involvement of the cortico–basal ganglia–thalamocortical loop in the pathogenesis of DYT3/XDP-TAF1 dystonia [18]. Goto et al. proposed a striatal compartmental hypothesis in the pathogenesis of dystonia, which may affect the cortico–basal ganglia–thalamocortical loop [19]. This hypothesis was derived from neuropathological findings in patients with DYT3/XDP-TAF1. According to this theory, striatal striosome compartment dysfunction may cause dystonia. Interestingly, the striosome compartment receives variable input from limbic-system-related cortices [20], which might account for psychiatric comorbidities. The striosome compartment could be a hub structure for interference between the motor and limbic functional loops, and we cannot deny the co-existence of organic and functional components in patients with dystonia [21].

The efficacy of pallidal-DBS varies in secondary dystonia. Pallidal-DBS is highly effective for tardive dystonia, even in the long term [22]. In contrast, some conditions, such as tonic-type dystonia, which is manifested in cerebral palsy, showed relatively lower improvement with pallidal-DBS [23]. Recently, DBS of the deep cerebellar nuclei or superior cerebellar peduncle has been reported to be effective in patients with tonic-type dystonia [24,25,26]. In addition to the compartmental hypothesis, accumulating evidence suggests that cerebellar dysregulation might be a critical element in the genesis of dystonia [27]. It is still unclear whether both the cortico–ponto–cerebello–thalamocortical and cortico–basal ganglia–thalamocortical loops are necessary for dystonia genesis. The complicated mixture of several components might account for the phenomenological diversity of dystonia and make it difficult for clinicians and researchers to elucidate the underlying cause. Dystonia is still one of the most mysterious syndromes, and much remains to be elucidated for better treatment and outcomes.
